# Poor Work Ability Is Associated with Workplace Violence in Nurses: A Two-Wave Panel Data Analysis

**DOI:** 10.3390/ijerph21091118

**Published:** 2024-08-24

**Authors:** Nicola Magnavita, Igor Meraglia

**Affiliations:** School of Occupational Health, Department of Life Sciences and Public Health, Università Cattolica del Sacro Cuore, 00168 Roma, Italy; meraglia.igor93@gmail.com

**Keywords:** ableism, ageism, disability management, health surveillance, health promotion, injury, social support, psychosocial stress, longitudinal study, bullying

## Abstract

Healthcare personnel must deal with two problems of growing importance: violence in the workplace and the loss of work ability due to the aging of the workforce. Our objective was to evaluate, with a two-wave perspective design, the relationships of work ability, social support, and occupational stress with workplace violence in nurses. In an Italian public health company, we asked nurses to self-assess their work ability using the Work Ability Index (WAI) and we analyzed the relationship between this indicator and the violence experienced in the previous and following years. A total of 321 out of 344 nurses (99.3%) participated. In a logistic regression model, the WAI score was a significant protective factor for violence experienced in the previous year (OR = 0.94 CI95% = 0.90; 0.98 *p* < 0.01) and in the following year (OR = 0.88 CI95% = 0.84; 0.92 *p* < 0.01). In a hierarchical logistic regression model, social support acted as a protective factor (OR = 0.87 CI95% = 0.79; 0.95 for violence experienced in the previous year), while occupational stress was a significant determinant of the risk of aggression (OR = 3.65 CI95% = 1.90; 7.03 in the previous year, OR = 3.54 CI95% = 1.801; 6.947 in the following year). The difficulties that nurses encounter in carrying out their growing work demands in an environment that is not promptly adapted to their changing physical and mental states can lead to an increased risk of violence. Prevention of workplace violence should include organizational and ergonomic measures that reduce stress and increase staff support and work ability.

## 1. Introduction

Workplace violence (WV) is a problem of considerable importance for healthcare workers (HCWs). Many studies have investigated this widespread phenomenon, its high prevalence in many occupational sectors, and the consequences endured by workers who are victims and witnesses of WV. Healthcare is the sector with the highest rate of non-fatal assaults. A meta-analysis of 253 studies involving a total of 331,544 participants that was performed before the COVID-19 pandemic indicated that 61.9% of HCWs had been exposed to some form of WV, as 42.5% reported exposure to non-physical WV and 24.4% had experienced physical WV in the previous year [1]. In the United States, the pooled rate of WV against nurses increased from 30% between 2000 and 2004 to 43% between 2020 and 2022 [2], with higher prevalence rates in emergency departments [3,4] and psychiatric mental health settings [5]. The COVID-19 pandemic changed working conditions and consequently aggression rates. The prevalence of WV during the first pandemic wave was lower compared to the period prior to the pandemic; however, it was still significant [6]: over 42% of HCWs had experienced violence [7]. The mid- to late-pandemic period witnessed an increase in physical and verbal violence prevalence, alongside a rise in legal litigation against HCWs [8]. While the verbal violence rate was equal in nurses and doctors, nurses were subjected to more than double the rate of physical violence than physicians [9,10]. Nurses are frequently victims of aggressive actions perpetrated by patients and visitors, but also by colleagues and superiors. Bullying against nurses takes on different forms in different cultures and occupational settings, but it is very widespread [11]. Inter-nurse horizontal violence is reported by over 33% of workers [12].

Copious studies on the effects of WV have enabled us to obtain valid evidence. Longitudinal studies in HCWs indicate that WV is consistently associated with poorer mental health [13,14]. Nurses experiencing WV suffer high levels of stress [15] and have 2.13- and 2.25-fold higher odds of reporting post-traumatic stress disorder and burnout than their non-exposed colleagues [16]. Poor training is one of the factors that predisposes nurses to experience violence in psychiatric settings [17]. It is important to observe that the relationship between WV and stress is reciprocal: workers who experience WV have increased work stress levels, and those who report high stress and low social support are prone to WV in subsequent years [18,19]. In more general terms, workers’ experience of occupational stress may make them more vulnerable to third-party violence. In hospitals, the highest rates of WV can be observed in situations associated with the highest level of work-related stress; social support is a protective factor [20].

Absence due to illness may be a consequence of the violence experienced. The literature indicates that WV exposure is associated with sickness leave [21]. In several studies, high rates of WV are associated with high absenteeism [22,23]. Lack of workplace social support is an independent risk factor for long-term absences associated with WV in Swedish and Danish cohort studies [24]. Once again, the relationship between WV and absenteeism is reciprocal. A meta-analysis showed that employees exposed to WV have a 26% higher risk of experiencing sickness absence, and that workers who have taken extended periods of sick leave have a greater possibility of subsequently encountering WV [25]. Social support reduces absenteeism in HCWs exposed to violent events [26]. On the contrary, lateral violence in nurses increases the risk of abandoning the profession [27].

Experiencing WV is also associated with work fatigue. Cross-sectional studies showed that nurses exposed to WV in emergency and first-aid services reported high levels of fatigue that impacted negatively on their personal lives, impaired patient care, and produced a toxic environment in the unit. In this degraded situation, lateral violence was both the source and the result of mental and emotional exhaustion [28]. Among workers from a psychiatric hospital, who reported regular exposure to social shaming and bullying by patients, WV was significantly associated with lower professional performance [29]. Emotions resulting from violence may influence the behaviour of HCWs; for example, exposure to verbal violence from patients increased the use of restraint and seclusion on the part of HCWs in a psychiatric hospital [30]. Moreover, the consequences of violence may trigger a spiral of violence.

Since exposure to WV can influence behaviour, work style, fatigue, stress, and sickness leave, it is important to study the relationship between violence and work capacity. Work ability (WA) is defined as a combination of occupational competence, the state of health needed to be competent, and the occupational skills needed to manage the work tasks in a reasonably safe environment [31]. Working capacity is closely linked to a person’s age and this makes it important to control this variable in healthcare personnel who are subject to progressive aging in all countries of the world [32,33,34,35,36,37]. The aging process and seniority of nurses negatively affect their ability to work [38] and this has prompted health promotion programs to counteract the effects of aging [39].

To measure work ability, authors have generally adopted the Work Ability Index (WAI) [40], a questionnaire created by Finnish researchers in the late 1990s and which is used all over the world, especially in healthcare workers [41], probably because hospital nurses have a high prevalence of inadequate work ability, and this can affect the treatment of patients [42]. In fact, it has been observed that the level of WAI in nurses is inversely correlated with errors and cognitive failure [43]. A low level of WAI has been associated with professional factors very common in nursing staff, such as stress [44,45,46] and night and precarious work [47], or with health problems, such as musculoskeletal disorders [48], a sedentary lifestyle [49], unhealthy diet and smoking [50], obesity [51], cardiovascular diseases [52], chronic diseases [53], and cancer [54]. Unfortunately, there are few longitudinal studies on the relationships between work ability and violence. The available studies of a cross-sectional nature indicate that the two variables are inversely associated, that is, increasing values of one variable correspond to decreasing values of the other variable. Most authors have interpreted the association observed in cross-sectional studies as evidence that violence reduces work capacity. For example, exposure to WV was considered responsible for the decline in the WAI of emergency physicians [55], social assistance workers [56], nurses [57,58], and mixed categories of HCWs [59].

Many researchers claim that cross-sectional studies do not allow us to infer causality, and this applies even more for a variable, such as violence, which has been shown to have reciprocal relationships with stress, absences, and fatigue, all factors linked to WA. Moreover, a careful examination of the characteristics of the WAI questionnaire suggests that these interpretations may be partial. The WAI is made up of seven dimensions that investigate chronic pathologies, occupational demands and individual resources [60] that contribute to compiling two main components, broadly described as “ill-health-related ability” and “subjectively estimated work ability”, respectively [61]. The first component collects information on illnesses diagnosed by a doctor, their impact on work capacity, absences due to illness, and the future occupational outlook. The second factor contains a self-evaluation of current working capacity compared to maximum working-life capacity, and an evaluation of the worker’s resources and their effectiveness compared to the physical and psychological demands of the job. It is plausible that many chronic pathologies pre-exist the act of violence and can be modified by WV to only a limited extent. Moreover, the reduction in work ability compared to the maximum possessed in life is considered an effect of aging rather than of trauma. When evaluating the association between WV and WA in a cross-sectional study, the possibility that reduced working capacity pre-existed and may have favored acts of violence must not be overlooked. 

To explore this possibility, using a short perspective design that measured exposure to violence over two successive years, we examined a sample of nurses from a public healthcare company to evaluate whether work ability can influence exposure to violence. In our study we tested the following hypotheses:

1. The WAI score is a significant predictor of the risk of aggression in nurses.
2. Social support is a protective factor against violence.
3. Occupational stress is a predisposing factor of aggression.

## 2. Materials and Methods

### 2.1. Population and Design of the Study

In European countries, workers who are exposed to occupational risks are subjected to a health surveillance program that includes recurrent medical examinations in the workplace. Following these check-ups, and after taking occupational risks into consideration, the doctor decides about the worker’s suitability for the job. One of the pieces of information that is routinely collected during these medical examinations is whether the worker has been exposed to violence in the previous year.

This study adopted a two-wave perspective model, in which work ability was measured at a single point in time, while WV was measured twice, namely at baseline and at follow-up, one year later. The difference with a classic longitudinal design is that in this study, only violence was measured twice, because it is assumed that working capacity does not change rapidly from one year to year for workers who continue the same task. Unlike what happens in repeated cross-sectional studies, in which the same information is asked to an independent sample at each wave, in this design the same individuals were asked the same information at multiple time points, which enable researchers to make a causal inference.

In an Italian public health company of the Lazio region, nurses received an invitation to evaluate their work capacity by filling in a form prior to their routine medical examination. Participation was free, unincentivized, and unrelated to the determination of their fitness for the job. A total of 321 of the 344 nurses who were invited to participate agreed to take part (participation rate 93.3%). 

The research was conducted in accordance with the Helsinki Declaration. In compliance with occupational medicine confidentiality principles and the ICOH code of ethics [62], participants granted their informed consent by signing a personal health document, thereby authorizing analysis of their personal data and agreeing to the dissemination of the results in an aggregate, anonymous form. The project was approved by the Catholic University Ethics Committee (ID 3008, 16 April 2020). 

### 2.2. Questionnaire

At the baseline, workers were invited to self-assess their working capacity using the Italian version [63] of the Work Ability Index (WAI) [40]. The WAI consists of a set of questions that consider the worker’s resources, health, and the physical and mental demands of their job. A total of 7 dimensions are used to grade the responses, resulting in a score ranging from 7 to 49. The WAI score can, therefore, be used as a continuous variable, with higher scores corresponding to greater work ability. The reliability of the questionnaire in this present study (Cronbach’s alpha) was 0.690. In the various studies conducted with this tool, it ranged from 0.54 to 0.83 [64,65,66,67,68,69].

In the public health company where this study was performed, the experience of violence in the workplace is recorded every year using four questions taken from the Arnetz Violent Incident Form (VIF) [70]. One question is used to measure physical violence in the workplace: “Have you been the victim of a physical attack while at work in the last 12 months? When we refer to a physical assault, we mean any attack—with or without weapons—that has the potential to cause bodily harm”. Similarly, questions with “yes” or “no” answers are used to investigate threats (“A threat refers to the intention of causing physical damage”) or harassment (“harassment is any annoying or unpleasant word, attitude or action that creates a hostile work environment,”). The fourth item involves indicating the perpetrator of the violence. At baseline, workers responded to questions about violence they had experienced in the previous year. One year later, in the next annual visit, workers responded to the same questions related to the year that had passed since the WAI was measured.

Workplace social support [71] was measured by means of six questions in the Italian version [72] of the demand–control–support questionnaire, based on Karasek’s job strain model [73]. Each question (e.g., “There is a quiet and pleasant atmosphere at my place of work”) had four possible answers rated on a score from 1 = “It’s never like that, I don’t agree at all” to 4 = “It’s exactly like that, I completely agree”. The final score varies from 6 to 24; higher values indicate greater social support. The reliability of the questionnaire in this study (Cronbach’s alpha) was 0.785.

Work-related stress was measured with the questionnaire derived from the Italian version [74] of Siegrist’s model [75]. The questionnaire is based on a 4-point Likert scale and consists of 3 questions for the Effort scale ranging from 3 to 12, and 7 questions for the Reward scale, ranging from 7 to 28. Stress, defined as the imbalance between effort and rewards (effort/reward imbalance, ERI), is measured as the weighted ratio between the two variables. The alpha of the Effort scale was 0.748, while the alpha of the Reward scale was 0.608.

### 2.3. Statistics

We analyzed the prevalence of workers who complained of having been exposed to different forms of violence in the two years of observation. We then studied the distribution of the variables of interest by using the Kolmogorov–Smirnov and Shapiro–Wilk tests. 

Firstly, we verified that the variables of interest (gender, age, WAI, support, and ERI) were correlated with each other by examining the Pearson correlation coefficients for parametric variables and Spearman correlation coefficients for non-parametric variables.

We then built some logistic regression models by taking each of the forms of violence reported by the nurses as a dependent variable, the WAI score as an independent variable, and age and sex as covariates. Finally, we built a hierarchical logistic regression model for each type of WV, first introducing the Support variable, and then using the work stress (ERI) variable as a covariate.

The IBM/SPSS Statistics for Windows software, version 28.0 (Armonk, NY, USA: IBM Corp), was used for these analyses.

## 3. Results

The study was conducted on 321 hospital nurses (72 male, 22.4%; 249 female workers, 77.6%). Mean age was 41.6 ± 13.0 years.

The average score of the WAI questionnaire was 40.1± 5.7 points; median value 41. The distribution of scores was non-normal (Kolmogorov–Smirnov test 0.141, *p* < 0.001; Shapiro–Wilk test 0.924, *p* < 0.001). Female workers had a slightly worse WAI score than males. Mean values did not reveal a significant gender difference (39.9 ± 5.5 in female vs. 40.9±6.1 in male workers, Student’s t = 1.39, *p* = 0.187); however, the comparison of medians demonstrated that work ability in women was significantly lower than in men (Mann–Whitney U Test *p* < 0.05). The WAI score was inversely correlated with age (Spearman’s rho = −0.182 *p* < 0.001). 

At the baseline, there were 40 workers (12.5%) who reported having experienced at least 1 episode of physical aggression in the previous year. A total of 57 nurses (17.8%) reported having been exposed to threats; 57 (17.8%) reported harassment. Overall, 96 people (29.9%) reported having been exposed to violence in the previous year. The main perpetrators of the assaults were patients (49.5%), visitors (26.8%), colleagues (22.7%), and, in one case only, a person unrelated to the work environment. At follow-up, the percentage of nurses who reported experiencing physical aggression in the previous year was slightly lower (36, 11.2%), while 53 had been subjected to threats (16.5%) and 75 (23.4%) reported harassment. Overall, there were 86 victims of violence (26.8%).

In order to evaluate the first hypothesis, i.e., whether the WAI score can be considered a predictor of violence, we built logistic regression models, adjusted for age and sex, using the occurrence of the different forms of violence as the dependent variable and the WAI score as the predictor. In these models, the WAI score at the baseline was a significant protecting factor for threats, harassment, and any type of violence experienced in the previous year (Table 1), and for WV experienced in the following year (Table 2).

To test the second hypothesis, that social support can protect against violence, and the third hypothesis, that stress promotes violence, we used hierarchical logistic regression analyses to evaluate the impact of workplace social support and stress on WV. Support exerted a modest protective effect on harassment but did not have a significant relationship with physical violence. Occupational stress was significantly associated with violence in all models (Table 3 and Table 4). The impact of work ability on the prevalence of different forms of violence in the following year remained significant even after the inclusion of social support and ERI in the hierarchical multivariate models.

## 4. Discussion

This study confirms that violence is a problem that nurses are forced to deal with on a daily basis. In the two years observed in our study, approximately one in four workers experienced at least one episode of physical or verbal violence. By asking workers during their annual medical examination if they had been victims of violence in the previous year, some episodes emerged which would otherwise have been neither reported nor recorded, demonstrating that health surveillance of workers can provide a contribution to the assessment of a risk that would otherwise remain unacknowledged.

Knowing the extent of a phenomenon and its causes is the basic requirement for controlling and preventing it. For this reason, we used a perspective method to investigate the possibility that violence is at least partly determined by reduced work ability. The fact that WAI score and risk of violence were inversely associated has been known for some time [55,56,57,58,59]. The authors interpreted this association as evidence that workplace violence causes a reduction in work ability. Our study gives greater weight to the reverse hypothesis, that poor work ability may increase the risk of suffering physical or verbal violence. This association may occur because workers at their full physical and mental capabilities are better able to manage situations that could lead to violent events. A similar point of view emerges from the Stockholm Public Health Cohort Study, which demonstrates that poor ability at baseline led to an increased risk of psychological distress at follow-up [76]. Similarly, poor work ability at baseline in our study led to high levels of violence experienced in the following year. It is important to remember that the Swedish study has a point of view opposite to that of previous longitudinal studies, which had stated that occupational stress causes a reduction in work ability [77,78]. The relationship between work ability and stress, as between work ability and violence, is probably reciprocal. Investigating the complexity of the relationship is essential to correctly plan prevention.

In this study, reduced work ability was associated with aggression experienced in the previous year and in an even more significant way with violence reported in the year following the WAI measurement. Although this observation, which was based on a limited sample, must be interpreted with caution, it might indicate that a reduced working capacity may gradually act as a promoter of violence because difficulty in providing the services required by the work task may induce a negative emotional state in healthcare workers that impairs their availability towards the requests of patients and visitors and consequently triggers incivility. Furthermore, workers with poor work ability may struggle to de-escalate violence. These could be some reasons why workers with low WAI have an increased risk of violence. Both these possible triggers of violence are linked to physical or mental skills that workers deem inadequate for their work tasks. However, another possible trigger of violence against nurses could be the perception of their vulnerability.

The factors that reduce the self-assessment of work ability made with the WAI are mainly chronic diseases and aging [79,80,81,82]. There is considerable evidence to suggest that disabled or elderly people are often particularly vulnerable to bullying and violence. Ableism (discrimination against disabled people) is an increasingly frequent occupational phenomenon [83] that directly affects the healthcare professions [84] and generates discrimination against HCWs [85,86]. Medical and nursing students are among the most frequent targets of discrimination [87]. A more modern approach in the medical field should allow for the inclusion of healthcare personnel with disabilities [88] but this does not always occur. Although policies and interventions have been developed to induce employers to manage disabilities correctly [89,90], these are still far from achieving their aims. A disabled person still undergoes stigma for being considered guilty of not carrying out his/her job. Perhaps this may explain why, when analyzing cross-sectional studies in WA and WV, authors have not explored the possibility that reduced work capacity may be among the causes of aggression. It is feared that the victim of the attack could be considered guilty of the attack itself. But this stems mainly from the idea that disability is a fault, and that the worker must adapt to the job, no matter how much effort it requires. On the contrary, it is the job that should be adapted to the worker, enhancing his/her residual work capabilities, but the literature demonstrates how far we are from applying this principle. The findings from a 2014 census of the Canadian federal public service revealed that disability was significantly associated with increased odds of workplace harassment and discrimination [91]. Discriminatory phenomena and WV also emerged from the Panel Survey of Employment for the Disabled 2016–2018 in South Korea [92], as well as from the Household, Income, and Labour Dynamics in Australia (“HILDA”) survey [93]. The same happens with aging: older nurses are often the targets of incivility in the workplace. The health and well-being of older nurses, as well as their ability to continue practicing, may be impacted by prevalently negative opinions and beliefs about their ability and skill in their occupational environments [94]. Therefore, we can affirm that the association between WA and WV observed in various studies should be interpreted primarily as a tendency to violence in workers who are unable to satisfy work demands. To prevent ageism and ableism in the nursing profession, work environments must foster a positive attitude to aging and disability by promoting meaningful relationships and an inclusive atmosphere. 

Evidence of the effect of WA on WV must not cause us to forget that, arguably, the relationship between WA and WV could be a reciprocal relationship. A study conducted with the Nurses Work Functioning Questionnaire [95], a tool specifically designed to investigate the work ability of nurses, demonstrated that exposure to violence does not modify the sub-scales relating to cognitive aspects of task execution, errors causing incidents at work, and avoidance behaviour, but is associated with conflicts with colleagues, impaired contact with patients and their families, and a lack of energy and motivation [96]. Therefore, violence does not affect cognitive abilities and professional skills, but profoundly disturbs work behaviour. Prolonged exposure to WV damages relationships between workers and increases occupational stress, which in the long term may also lead to a reduction in the individual resources that sustain adequate work ability.

This study showed that good relationships with colleagues and superiors, i.e., workplace social support, can help reduce the risk of harassment but that they have little effect on physical aggression. Occupational stress is associated with manifestations of violence. There is a close reciprocal relationship between violence and stress, as well as between violence and social support [18,19]. The repetition of violent episodes induces chronic stress and reduces the perception of social support, and this predisposes workers to new episodes of violence. Workplace support is of particular importance in moderating the effects of the interaction between work ability and violence. Nursing students attribute the vertical violence they experience during clinical work primarily to a lack of information and guidance [97], while, on the contrary, students exposed to intense clinical stress but with high social support from teachers obtain optimal results [98]. Verbal and psychological violence also has serious effects on nursing students who complain about high job strain, low social support, and low organizational justice [99] that produce a negative effect on their professional commitment [100]. Workplace violence in HCWs may be associated with burnout and the intention to quit [101], although this relationship can be mediated by work ability [102]. 

This study has the merit of having addressed the relationship between work ability and workplace violence in an original way. The judgment that each worker has of their own working capacity (WAI) in relation to their state of health and the demands of the job was considered an independent variable, like two variables (stress and social support) whose effect on risk of violence had already been demonstrated by previous studies. The violence experienced in the year preceding the WAI survey, and that suffered in the following year, were placed as dependent variables in linear regression models. This approach arises from the fact that violence should not be considered an unpredictable and unavoidable factor, but rather a working circumstance to be measured and controlled. Statistical analyses have shown that poor work ability is associated with a high risk of violence. This result offers a new perspective for the prevention of violence at work, through the improvement of work ability.

The strengths of the study were the high participation of workers, the simple epidemiological design, and the very low cost, because all the data were obtained from the health surveillance of workers, which is mandatory by law. Other corporate occupational health services will be able to easily duplicate this study.

By showing how poor work ability can be associated with exposure to violence, this study deserves credit for shedding light on a topic that has so far attracted little research. However, it has many limitations. The first is that it was conducted on a single healthcare company, thus restricting the possibility of extending our results to other work situations, even though there were no obvious differences between the nurses of the public company examined here and other nurses. Another limitation is the shortness of the prospective observation period. A cohort study conducted over a period of several years could help reveal the possible reciprocal relationships between violence and work ability. To date, the topic has only been addressed by cross-sectional studies that cannot infer the causality of the observed association. Having measured work ability at a single point in time prevented us from evaluating any, even if unlikely, variations in this variable. Finally, a further limitation is the reliance of the study on subjective data. Nevertheless, recording experiences of violent episodes during an annual medical examination by the occupational health physician provides more sensitive data than an analysis of injury official reports, which include only the worst episodes of physical violence. Moreover, it is much more accurate than the application of algorithms that have not been scientifically validated [103]. Better knowledge of the relationship between work ability and violence can be obtained with well-designed, long-term longitudinal studies.

## 5. Conclusions

Our study confirmed the three hypotheses underlying this research. Work ability is a protective factor towards violence at work. Social support at work can act as a protective factor, while occupational stress is significantly associated with high odds ratios of violence. Correctly framing the relationships between work ability and violence is the first step to preventing violence at work. If the violence arises from discrimination against the elderly or disabled worker, action must be taken on these points. Similarly, if the violence originates from poor training of workers in WV de-escalation procedures, they can be conveniently trained [104]. The prevention of violence in the workplace should not be separated from ergonomic and organizational interventions to improve work capacity and reduce occupational stress.

## Figures and Tables

**Table 1:** Association of WAI score with exposure to workplace violence experienced in the previous year. Logistic regression analysis models adjusted for age and sex.

Type of Violence	Odds Ratio *	Confidence Intervals 95%	*p*
Physical	0.945	0.891; 1.003	0.062
Threats	0.943	0.899; 0.990	0.018
Harassment	0.941	0.896; 0.988	0.014
Any type of violence	0.942	0.903; 0.983	0.006

* Adjusted for age and sex.

**Table 2:** Association of WAI score with exposure to workplace violence in the following year. Logistic regression analysis models adjusted for age and sex.

Type of Violence	Odds Ratio *	Confidence Intervals 95%	*p*
Physical	0.890	0.840; 0.942	0.001
Threats	0.887	0.842; 0.933	0.001
Harassment	0.885	0.844; 0.929	0.001
Any type of violence	0.880	0.839; 0.923	0.001

* Adjusted for age and sex.

**Table 3:** Association of WAI score, social support, and occupational stress with exposure to workplace violence in the previous year. Hierarchical logistic regression analysis.

Type of Violence		Model I * OR (CI95%)	*p*	Model II ** OR (CI95%)	*p*
Physical	WAI	0.952 (0.896; 1.012)	0.114	0.980 (0.917; 1.047)	0.551
	Support	0.944 (0.832; 1.070)	0.367	1.012 (0.876; 1.168)	0.875
	ERI	-	-	3.903 (1.719; 8.861)	0.001
Threats	WAI	0.950 (0.903; 1.000)	0.049	0.975 (0.923; 1.031)	0.373
	Support	0.951 (0.855; 1.059)	0.361	1.020 (0.904; 1.151)	0.743
	ERI	-	-	3.867 (1.914; 7.812)	0.001
Harassment	WAI	0.963 (0.914; 1.014)	0.151	0.994 (0.938; 1.053)	0.843
	Support	0.844 (0.757; 0.940)	0.002	0.899 (0.800; 1.012)	0.077
	ERI	-	-	4.739 (2.294; 9.790)	0.001
Any type of violence	WAI	0.960 (0.917; 1.004)	0.074	0.984 (0.937; 1.033)	0.512
	Support	0.868 (0.790; 0.953)	0.003	0.919 (0.831; 1.016)	0.097
	ERI	-	-	3.655 (1.900; 7.030)	0.001

* Adjusted for age, sex, and social support. ** Additionally adjusted for ERI.

**Table 4:** Association of WAI score, social support, and occupational stress with exposure to workplace violence in the subsequent year. Hierarchical logistic regression analysis.

Type of Violence		Model I * OR (CI95%)	*p*	Model II ** OR (CI95%)	*p*
Physical	WAI	0.889 (0.837; 0.944)	0.001	0.902 (0.847; 0.961)	0.001
	Support	1.015 (0.889; 1.159)	0.828	1.068 (0.922; 1.237)	0.382
	ERI	-	-	2.495 (1.088; 5.724)	0.031
Threats	WAI	0.894 (0.847; 0.943)	0.001	0.908 (0.858; 0.961)	0.001
	Support	0.946 (0.845; 1.059)	0.336	1.008 (0.890; 1.143)	0.897
	ERI	-	-	3.296 (1.581; 6.873)	0.001
Harassment	WAI	0.895 (0.851; 0.941)	0.001	0.912 (0.865; 0.962)	0.001
	Support	0.917 (0.827; 1.016)	0.096	0.991 (0.884; 1.110)	0.872
	ERI	-	-	4.587 (2.261; 9.304)	0.001
Any type of violence	WAI	0.889 (0.846; 0.934)	0.001	0.904 (0.858; 0.952)	0.001
	Support	0.921 (0.834; 1.017)	0.104	0.982 (0.881; 1.094)	0.741
	ERI	-	-	3.537 (1.801; 6.947)	0.001

* Adjusted for age, sex, and social support. ** Additionally adjusted for ERI.

## Data Availability

Anonymized data can be obtained upon request.
